# Economic burden of varicella in Europe in the absence of universal varicella vaccination

**DOI:** 10.1186/s12889-021-12343-x

**Published:** 2021-12-21

**Authors:** Manjiri Pawaskar, Estelle Méroc, Salome Samant, Elmira Flem, Goran Bencina, Margarita Riera-Montes, Ulrich Heininger

**Affiliations:** 1 Merck & Co., Inc. Center for Observational and Real-World Evidence, 2000 Galloping Hill Road, Kenilworth, NJ 07033 USA; 2 P95 Epidemiology and Pharmacovigilance, Koning Leopold III laan 1, 3001 Leuven, Belgium; 3 MSD (Norge) AS, Postboks 458 Brakerøya, 3002 Drammen, Norway; 4 MSD, Calle de Josefa Valcárcel, 38, 28027 Madrid, Spain; 5 University of Basel Children’s Hospital (UKBB), Spitalstrasse 33, 4056 Basel, Switzerland; 6 Faculty of Medicine, University of Basel, Klingelbergstrasse 61, 4056 Basel, Switzerland

**Keywords:** Varicella, Economic burden, Costs, Europe

## Abstract

**Background:**

Though the disease burden of varicella in Europe has been reported previously, the economic burden is still unknown. This study estimated the economic burden of varicella in Europe in the absence of Universal Varicella Vaccination (UVV) in 2018 Euros from both payer (direct costs) and societal (direct and indirect costs) perspectives.

**Methods:**

We estimated the country specific and overall annual costs of varicella in absence of UVV in 31 European countries (27 EU countries, plus Iceland, Norway, Switzerland and the United Kingdom). To obtain country specific unit costs and associated healthcare utilization, we conducted a systematic literature review, searching in PubMed, EMBASE, NEED, DARE, REPEC, Open Grey, and public heath websites (1/1/1999–10/15/2019). The number of annual varicella cases, deaths, outpatient visits and hospitalizations were calculated (without UVV) based on age-specific incidence rates (Riera-Montes et al. 2017) and 2018 population data by country. Unit cost per varicella case and disease burden data were combined using stochastic modeling to estimate 2018 costs stratified by country, age and healthcare resource.

**Results:**

Overall annual total costs associated with varicella were estimated to be €662,592,061 (Range: €309,552,363 to €1,015,631,760) in Europe in absence of UVV. Direct and indirect costs were estimated at €229,076,206 (Range €144,809,557 to €313,342,856) and €433,515,855 (Range €164,742,806 to €702,288,904), respectively. Total cost per case was €121.45 (direct: €41.99; indirect: €79.46). Almost half of the costs were attributed to cases in children under 5 years, owing mainly to caregiver work loss. The distribution of costs by healthcare resource was similar across countries. France and Germany accounted for 49.28% of total annual costs, most likely due to a combination of high numbers of cases and unit costs in these countries.

**Conclusions:**

The economic burden of varicella across Europe in the absence of UVV is substantial (over 600 M€), primarily driven by caregiver burden including work productivity losses.

**Supplementary Information:**

The online version contains supplementary material available at 10.1186/s12889-021-12343-x.

## Background

Varicella-zoster virus, from the α-herpesvirus family, causes varicella (or chickenpox) on primary infection and herpes zoster (HZ) (or shingles) upon reactivation. Varicella is a highly communicable disease, typically affecting children 2–8 years of age [1, 2]. Varicella is usually mild with an average incubation period of 14–16 days and characterized by an itchy vesicular rash accompanied by fever and malaise. In some cases, serious complications such as superinfection of skin lesions or disseminated infections such as pneumonia and encephalitis may occur. These complications may require hospitalization, and may in rare instances, lead to long-term sequelae or death [2, 3]. Although the risk of complications is higher in infants, adults, pregnant women and immunocompromised persons, most varicella-related complications occur among immunocompetent children with no underlying medical conditions [3].

Varicella vaccines are safe and efficacious in preventing varicella, particularly severe varicella [3]. In the absence of universal immunization, the disease burden of varicella would be substantial with a total of 5.5 million (95% CI: 4.7–6.4) cases occurring annually across Europe, with the majority occurring in children younger than 5 years. Annually, this would result in 3–3.9 million varicella patients consulting a primary care physician, 18,200–23,500 hospitalizations, and 80 (95% CI: 19–822) deaths [4]. Because varicella-zoster virus is highly contagious, infection control practices in most countries include isolation of patients until lesions are crusted and dry, which usually takes about 7 days. The associated work absenteeism and productivity loss among caregivers and adult cases is expected to result in considerable economic impact at the population level, especially in the countries with high annual varicella case counts owing to large population [5–9].

In 2014, The World Health Organization (WHO) recommended that countries which are able to sustain a vaccine coverage of at least 80% should consider introducing varicella into routine childhood immunization programs [10]. Yet, as of 2021, less than 50% of European countries have implemented Universal Varicella Vaccination (UVV) or have national recommendations for universal vaccination [11–13]. Identifying and measuring the costs attributable to varicella is important for decision-making about vaccination introduction [14]. Thus, we aimed to quantify the overall, as well as country-specific economic burden of varicella in Europe. Building on previous research on the burden of disease [4] in countries without universal immunization, we evaluated the varicella-associated costs from payer (direct costs) and societal perspectives (direct and indirect costs) in 31 European nations.

## Methods

This study was conducted in three successive steps: 1. A systematic literature review (SLR) was conducted to obtain unit cost and healthcare resource utilization parameters; 2. Disease burden was updated using published incidence rates and 2018 population estimates; and 3. Economic burden was estimated by combining burden of disease and unit cost/utilization parameters. All costs were estimated for the 2018 European population (for 27 European Union countries, plus Iceland, Norway, Switzerland and the United Kingdom (UK)) and were reported in Euros (year 2018).

### SLR for unit cost and utilization parameters

We conducted a SLR for peer-reviewed studies published between 1 January 1999 and 15 October 2019 in any language to obtain country-specific unit costs per varicella case and healthcare resource utilization parameters. The database search was conducted in MEDLINE (via PubMed), EMBASE, NHS Economic Evaluation Database (NEED), Database of Abstracts of Reviews of Effectiveness (DARE), Research Papers in Economics (REPEC) and Open Grey. Data were also obtained from the WHO, European Center for Disease Prevention and Control (ECDC) and national public health institutes websites. Two types of outcomes were of interest:

1. Unit cost items per varicella case: including outpatient physician visits, emergency room (ER) visits, hospital visits (outpatient unit of hospital), hospitalizations (total or one-day), intensive care unit (ICU) stay, other healthcare professionals visits (physiotherapist, psychologist, specialized physician and other healthcare professional), over-the-counter (OTC) medications, prescription medications in outpatients (e.g. antiviral medication), tests and procedures in outpatients.
2. Per case resource utilization: including length of hospitalization, work days lost by caregiver for outpatient/inpatient cases, work days lost by adult outpatient/inpatient cases, utilization rate for ER visits, hospital visits, ICU stays, OTC medications, other healthcare professional visits, prescription medications and tests and procedures.

The SLR was conducted in accordance with the standards of the “Preferred Reporting Items for Systematic Reviews and Meta-Analyses” (PRISMA) guidelines and a flowchart was prepared to describe the process [15]. Titles and abstracts from the list of references were independently screened by MR and EM to identify studies that fulfilled the selection criteria. Studies were eligible for inclusion if 1) they concerned varicella-zoster virus primary infection (excluding studies on varicella-zoster reactivation alone), 2) provided data for at least one of the abovementioned outcomes of interest, 3) concerned data collected in a European country, and 4) were primary data (review publications were excluded although reference lists were screened to identify additional publications). Discrepancies were discussed and resolved without the need for a third reviewer. Subsequently, full-text eligibility was evaluated by the reviewers and relevant data extracted. The full search string used for the SLR is available in Additional file 1.

The extracted cost and utilization outcomes were summarized for each country. For countries with multiple entries per outcome, a range (min-max) was generated based on all values extracted. Values expressed in currency prior to 2018 were adjusted for inflation using the 2018 price index (Gross Domestic Product (GDP) price deflator) [16]. For countries for which no data was available for a given unit outcome, two different types of imputation values were generated: For utilization outcomes, we used the mean value from other countries with available data and for cost outcomes, we generated the input by weighting values by 2018 Purchasing Power Parities (PPP) [17]. Full details can be found in Additional file 2.

### Disease burden parameters

We calculated the mean (range: min-max) annual number of varicella cases and deaths, outpatient visits and hospitalizations in 2018 using previously reported data on varicella incidence rates in Europe before UVV [4]. We updated these incidence numbers stratified by country and age group (< 5, 5–9, 10–14, 15–19, 20–39, 40–64 years) with 2018 population estimates (Additional file 3).

### Total annual costs

Variables to be included in the model were chosen based on data availability, and information from at least five countries was required to include an outcome. The following outcomes were included: unit cost of outpatient visit/hospitalization/prescription medication/OTC medication and utilization (proportion) of prescription medication/OTC medication, length of hospital stay, and number of work days lost by caregiver/adult case. To account for the uncertainty of the input parameters, assuming that all values falling within the min-max range were equally probable, a uniform distribution (min-max) was assigned to each parameter in our model. The parameter distributions were then combined through stochastic modeling; a total of 10,000 iterations were ran, and the mean and min-max output of the output values were used to estimate the population-level direct costs (outpatient visits, hospitalizations, prescription/OTC medications) and indirect costs (work loss caregivers/patients/deaths) were estimated for the 31 European countries. The distribution of costs by country, age group and healthcare resource was described. Finally, the mean (direct/indirect/total) costs per varicella case were calculated by dividing the total annual costs by the number of annual varicella cases in Europe. In addition, sensitivity analysis was conducted by: 1. considering only countries without general recommendation for publicly funded varicella vaccination as of 2021 [13], 2. adjusting the unit costs of the tradeable health care costs (i.e. OTC and prescription medication) using exchange rates. The final cost model is described in Additional file 4.

## Results

**Fig. 1:**
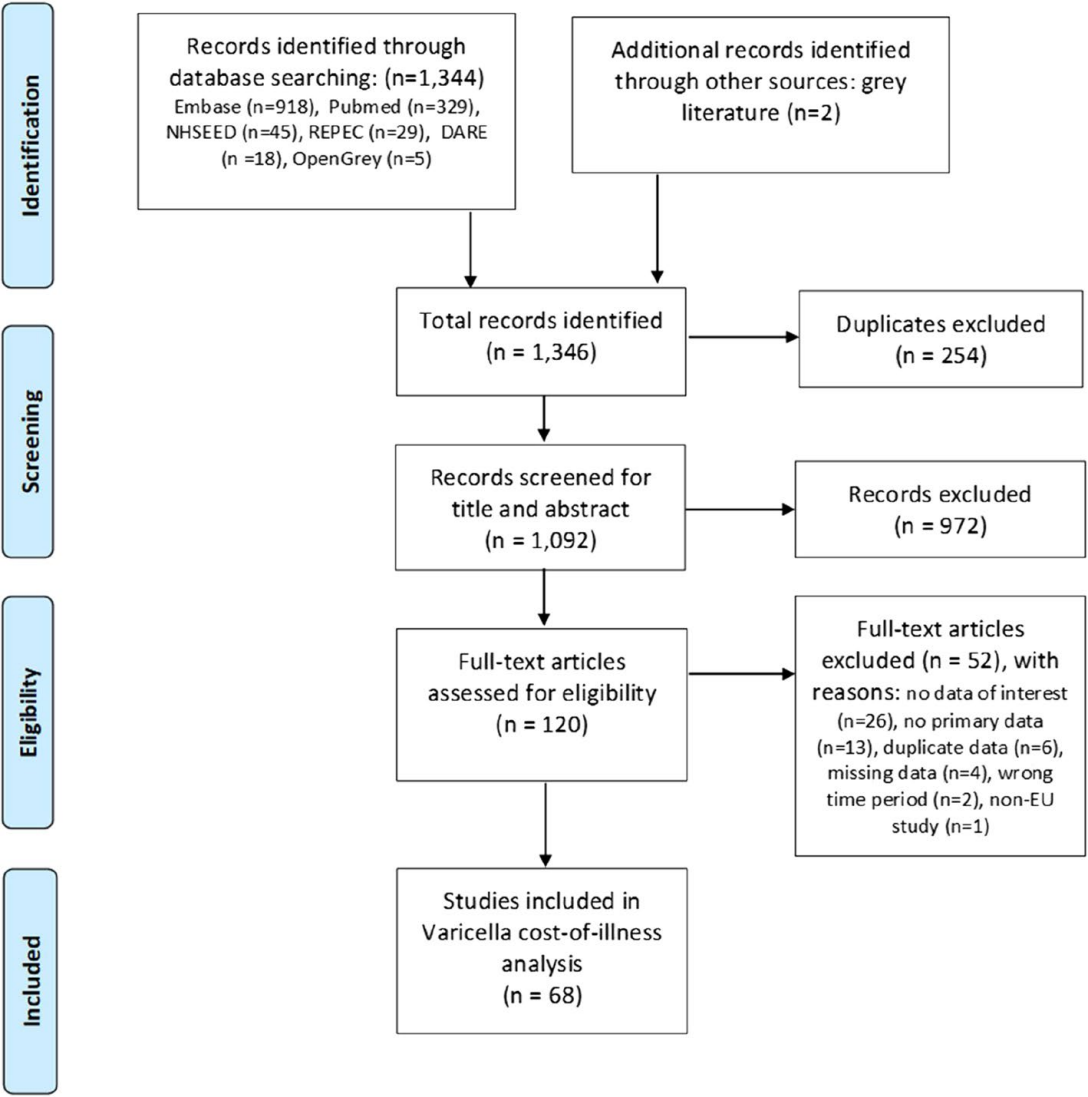
PRISMA Flow Diagram

As described in the PRISMA diagram (Fig. 1), 120 full-text articles were assessed for eligibility, of which 68 were selected for data extraction. The extracted data is described in Table 1. Hungary, Poland and Spain were the countries for which data was retrieved for the highest number of cost/utilization outcomes. In four countries- Austria, Cyprus, Finland and Iceland- no varicella unit cost/utilization data was retrieved.

**Table 1:** Data extraction summary of unit cost and health resource utilization items

Cost item	No.	Countries
Cost of physician visit	10	CH [18], FR [19–21],DE [5, 19],ES [8, 22–29],HU [30],IT [31, 32],NL [33],NO [9],PL [34], UK [35–37]
Cost of ER visit	4	ES [8, 23–25, 27],HU [30],NO [9],PL [34]
Cost of hospital visit	3	HU [30],NO [9],PL [34]
Cost of hospitalization	11	CH [18],BE [38],DE [5, 19, 39],ES [22–24, 26–28, 40–45],FR [20, 21, 46, 47],HU [30], IT [31, 32, 48, 49], NO [9], NL [33], PL [34],UK [35–37, 50]
Cost of ICU stay	4	DE [5, 39],ES [28],HU [30],IT [48]
Cost of OTC medications	7	CH [18], DE [5], ES [22–25, 29], HU [30],IT [32],PL [34],UK [35]
Cost of other health visit	2	HU [30],PL [34]
Cost of prescription medications	5	ES [23, 25],HU [30],IT [31, 32],PL [34],UK [35–37]
Cost of tests/procedures	4	CH [18],ES [45],HU [30],PL [34]
Health resource utilization item	**No. C**	**Countries**
Length of hospitalization	16	BE [38, 51],CH [52],CZ [53],DK [54],DE [55–57],EL [58],ES [22, 24, 25, 40–45, 59–62],FR [46, 47, 63–67],HU [30],IT [48, 49, 68, 69], NL [70],NO [71],PO [34, 72–74],RO [75],SE [76] UK [35, 36, 50]
No. Work days lost: caregivers (inpatient)	4	BE [38],HU [30],PL [34],UK [35]
No. Work days lost: caregivers (outpatient)	10	BE [38],FR [19, 46, 64],DE [5, 19, 57, 77],ES [8, 23, 25],HU [30],IT [31, 32, 78],NL [33, 70], PL [34],SE [76],UK [35]
No. Work days lost: adults (inpatient)	2	BE [38],UK [35]
No. Work days lost: adults (outpatient)	7	CH [18],DE [5, 19, 77],ES [8, 25],FR [19–21, 46, 64],IT [31, 49],NL [33],UK [35]
Utilization ER visit	4	ES [8],HU [30],PL [34],NL [79]
Utilization hospital visit	2	HU [30],PL [34]
Utilization ICU stay	2	HU [30], IT [48]
Utilization OTC medications	4	ES [24],HU [30],IT [32],PL [34]
Utilization other health visit	2	HU [30],PL [34]
Utilization prescription medication	5	ES [8, 80],HU [30],IT [31, 32, 81],NL [79, 82], PL [34]
Utilization tests/procedures	3	ES [8],HU [30],PL [34]

*Abbreviations*: *BE* Belgium, *BG* Bulgaria, *CH* Switzerland, *CZ* Czechia, *DE* Germany, *DK* Denmark, *EE* Estonia, *EL* Greece, *ER* Emergency Room, *ES* Spain, *FR* France, *HR* Croatia, *HU* Hungary, *ICU* Intensive Care Unit, *IE* Ireland, *Inpt* Inpatient, *IT* Italy, *LV* Latvia, *LT* Lithuania, *LU* Luxembourg, *MT* Malta, *NL* Netherlands, *NO* Norway, *OTC* over-the-counter, *PL* Poland, *PT* Portugal, *RO* Romania, *SE* Sweden, *SI* Slovenia, *SK* Slovakia, *UK* United Kingdom

The outcomes with sufficient data (i.e. > 5 countries with at least one data source) are described in Table 2, stratified by high income countries versus middle and low income countries. The updated 2018 number of annual varicella cases, deaths, outpatient visits and hospitalizations as well as the full extracted unit cost and utilization outcomes are available in Additional files 3 and 5, respectively.

**Table 2:** Descriptive statistics of extracted unit cost and utilization outcomes in high income (Belgium, Denmark, France, Germany, Netherlands, Norway, Spain, Sweden, Switzerland and UK) and low- and middle-income (Czechia, Greece, Hungary, Italy, Poland and Romania) countries

Unit parameter	Median	Min	Max
**a. High income countries**			
Cost of physician visit (EUR)	325.08	4.52 [29]	72.41 [22]
Cost of 1 day hospitalization (EUR)	554.36	128.85 [24]	1304.44 [9]
Length of hospitalization (days)	4.85	1.7 [67]	9 [43]
Cost of OTC medications (EUR)	14.34	2.05 [24]	15.86 [18]
Utilization OTC medications^a^ (%)	100	100 [24]	100 [24]
Cost of prescribed medications (EUR)	15.16	2.8 [35]	25.77 [23]
Utilization of prescribed medications (%)	70.3	47.7 [82]	100 [8]
Work lost by caregiver^b^ (days)	1.42	0.3^]^ [70]	6.6 [57]
Work lost by patient^b^ (days)	8.1	1.44 [18]	18.72 [5]
**b. Low- and middle-income countries**			
Cost of physician visit (EUR)	29.02	9.55 [30]	52.29 [34]
Cost of 1 day hospitalization (EUR)	155.35	97.03 [30]	629 [32]
Length of hospitalization (days)	5.89	3.6 [30]	7.9 [68]
Cost of OTC medications (EUR)	0.55	0.4 [30]	4.84 [32]
Utilization of OTC medications^a^ (%)	96	80 [34]	100 [32]
Cost of prescribed medications (EUR)	21.39	9.4 [34]	31.57 [30]
Utilization of prescribed medications (%)	72	9.3 [30]	100 [32]
Work lost by caregiver^b^ (days)	2.61	0.6 [31]	4.98 [78]
Work lost by patient^b^ (days)	6.8	2.6 [49]	11 [31]

^a^ available for 4 countries; despite the fact that the parameter was available for less than 5 countries (cf. selection criterion), it was decided to include it in the cost model because the associated parameter, cost of OTC medications, was already included

^b^ for outpatient cases

Overall annual total costs associated with varicella in Europe were €662,592,061 (Min-Max: €309,552,363–€1,015,631,760) (Table 3). Direct and indirect costs were estimated at €229,076,206 (Min-Max: €144,809,557–€313,342,856) and €433,515,855 (Min-Max: €164,742,806–€702,288,904). Average total cost per case was estimated at €121.45 (Min-Max: €56.74–€186.17), of which €41.99 (Min-Max: €26.54–€57.44) were direct costs and €79.46 (Min-Max: €30.20–€128.73) were indirect costs. Varicella deaths represented only a limited proportion of indirect costs (0.051%).

**Table 3:** Estimated annual direct and indirect costs (million €) associated with varicella in Europe in absence of UVV

Country		Total costs			Direct costs			Indirect costs		
	Annual Cases	Mean (M€)	Min (M€)	Max (M€)	Mean (M€)	Min (M€)	Max (M€)	Mean (M€)	Min (M€)	Max (M€)
Austria	87,629	9.44	7.76	11.11	3.55	3.06	4.04	5.88	4.70	7.07
Belgium	126,859	8.66	8.22	9.10	3.62	3.22	4.02	5.04	5.00	5.08
Bulgaria	68,333	2.61	2.16	3.05	1.35	1.16	1.54	1.25	1.00	1.51
Croatia	40,580	2.23	1.84	2.63	1.03	0.88	1.17	1.21	0.96	1.45
Cyprus	9126	0.65	0.56	0.74	0.26	0.23	0.28	0.39	0.33	0.46
Czechia	113,658	7.31	6.04	8.57	3.24	2.79	3.69	4.07	3.25	4.89
Denmark	63,557	7.23	6.39	8.07	2.58	2.36	2.80	4.65	4.03	5.27
Estonia	14,709	1.01	0.84	1.19	0.46	0.39	0.52	0.56	0.45	0.67
Finland^a^	56,373	5.09	4.50	5.67	2.08	1.87	2.29	3.01	2.62	3.39
France	794,533	174.58	49.37	299.79	41.40	26.12	56.69	133.18	23.25	243.10
Germany^a^	761,182	151.91	43.01	260.82	28.57	20.56	36.58	123.34	22.44	224.24
Greece^a^	104,441	8.79	6.84	10.73	3.56	2.93	4.19	5.23	3.91	6.54
Hungary^a^	96,068	4.03	3.24	4.81	0.96	0.80	1.12	3.07	2.44	3.69
Iceland^a^	4330	0.51	0.46	0.56	0.20	0.19	0.22	0.31	0.28	0.34
Ireland	63,328	7.74	6.87	8.60	2.10	1.94	2.26	5.63	4.93	6.33
Italy^a^	542,700	75.09	28.30	121.89	32.01	19.51	44.52	43.08	8.79	77.37
Latvia^a^	21,088	1.21	1.01	1.42	0.55	0.48	0.63	0.66	0.53	0.79
Lithuania	29,903	1.62	1.35	1.89	0.74	0.64	0.83	0.88	0.71	1.05
Luxembourg^a^	6479	0.98	0.87	1.09	0.22	0.20	0.24	0.75	0.66	0.84
Malta	4944	0.41	0.36	0.46	0.17	0.15	0.18	0.24	0.21	0.28
Netherlands	173,107	9.43	4.77	14.08	3.18	2.55	3.81	6.25	2.22	10.27
Norway	61,286	7.81	6.85	8.77	3.14	2.86	3.43	4.67	4.00	5.34
Poland	396,449	22.80	22.39	23.21	12.78	12.41	13.14	10.03	9.99	10.07
Portugal	89,252	6.67	5.79	7.54	2.70	2.44	2.96	3.97	3.36	4.58
Romania	200,340	4.55	4.44	4.65	2.48	2.38	2.57	2.07	2.06	2.07
Slovakia	58,699	3.40	2.85	3.95	1.45	1.27	1.63	1.95	1.58	2.32
Slovenia	21,331	1.75	1.52	1.99	0.60	0.54	0.66	1.15	0.98	1.33
Spain^a^	450,617	48.14	21.76	74.52	30.52	7.69	53.35	17.62	14.08	21.17
Sweden	120,826	15.11	13.18	17.03	4.53	3.96	5.09	10.58	9.22	11.95
Switzerland	85,150	8.69	7.74	9.64	3.71	3.41	4.01	4.97	4.32	5.62
United King.	788,581	63.15	38.24	88.05	35.31	15.79	54.84	27.83	22.45	33.21
TOTAL	**5,455,459**	**662.59**	**309.55**	**1015.63**	**229.08**	**144.81**	**313.34**	**433.52**	**164.74**	**702.29**

^a^UVV as of 2021

**Fig. 2:**
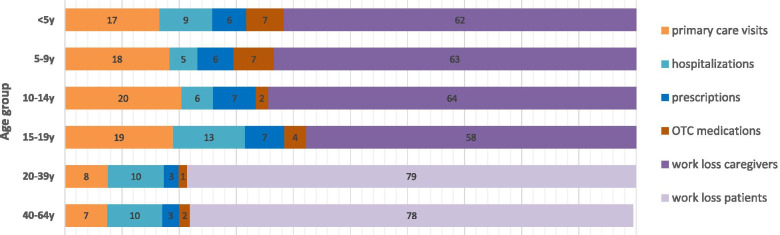
Distribution of age-specific varicella costs by healthcare resource [Proportion (%)]

**Fig. 3:**
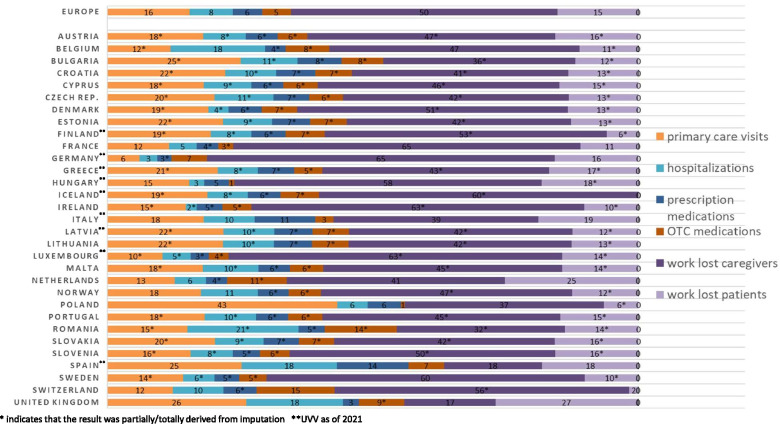
Distribution of country-specific varicella costs by healthcare resource [proportion (%)]

Almost half of the total costs were associated with cases in children below 5 years of age (Table 4), of which indirect costs due to work absenteeism among caregivers represented 61.65% (Fig. 2). Countries with the highest varicella-associated costs in the absence of universal vaccination were France and Germany (Table 3), which together accounted for 49.28% of total costs. These two countries and the UK also had the highest number of annual varicella cases (France: 794,533; Germany: 761,182; UK: 788,581). The distribution of total varicella costs by type of healthcare resource was similar across European countries (Fig. 3) with indirect costs due to work loss among caregivers or patients accounting for the largest proportion of costs in almost all countries. Among the direct cost components, the major cost drivers were primary care visits (16% of total costs, range 6% in Germany to 43% in Poland), followed by hospitalizations (8% of total costs, range 2% in Ireland to 21% in Romania).

**Table 4:** Estimated Distribution of varicella costs (€) in Europe by age group and health care resource in absence of UVV

Age group	Primary care visits	Hospitalizations	Prescriptions	OTC medications	Work loss caregivers	Work loss patients	Work loss deaths	Proportion of total cost
<5y	€ 51,939,569	€ 29,121,301	€ 18,648,121	€ 20,541,844	€ 193,314,712	NA	NA	47.32%
5-9y	€ 30,605,288	€ 8,138,576	€ 10,516,603	€ 11,741,757	€ 105,800,000	NA	NA	25.17%
10-14y	€ 7,830,211	€ 2,117,755	€ 2,853,437	€ 826,043	€ 24,659,029	NA	NA	5.78%
15-19y	€ 2,936,218	€ 1,940,903	€ 1,071,301	€ 585,661	€ 8,938,000	NA	NA	2.34%
20-39y	€ 7,259,423	€ 9,407,695	€ 2,517,461	€ 1,362,976	NA	€ 75,435,180	€ 57,955	14.49%
40-64y	€ 2,413,798	€ 3,123,022	€ 961,981	€ 615,262	NA	€ 25,146,631	€ 164,349	4.89%
Total	**€ 102,984,507**	**€ 53,849,251**	**€ 36,568,905**	**€ 35,673,544**	**€ 332,711,741**	**€ 100,581,811**	**€ 222,303**	**100.00%**

When the 9 countries with UVV were excluded from the analysis, the total costs were €366,839,010 (Min-Max: €199,562,734–€534,115,287). Adjusting the unit costs of the tradeable health care costs (OTC and prescription medication) using exchange rates instead of PPP resulted in a reduction of total costs of 662,375 EUR (0.1% of total costs).

## Discussion

The estimated economic burden of varicella in the absence of UVV across 31 European countries would be substantial, amounting to over €660 million annually in 2018 Euros. Of the total cost, 65% were attributed to work loss suggesting a significant societal burden and impact on productivity. To our knowledge, this study is the first study to systematically estimate the economic burden of varicella for individual European countries. The substantial costs associated with productivity losses estimated in our study are in line with findings from previous systematic literature reviews on varicella burden [3, 83]. Banz et al. [5] found that prior to routine vaccination implementation, in Germany, 82% of varicella-associated costs were attributable to work loss. Another study from the US conducted during the pre-vaccine era also concluded that costs associated with parental work absenteeism represented 95% of the total cost [84].

Primary care visits were the key driver among direct costs associated with varicella in almost all countries (46% of direct costs on average). It was previously shown that most varicella cases in Europe lead to a physician consultation (54%), whereas only a minority of cases (0.3%) are hospitalized [4]. This is consistent with the literature considering the relatively mild nature of the disease in young children.

The greatest share of costs was associated with cases in children younger than 5 years, reflecting that varicella incidence is highest in this age group [4], whereas higher rates of varicella-associated complications and hospitalization occurred in the 10–14 year and 20–39 year age groups.

The distribution of varicella costs by healthcare resource appeared to be generally similar across European countries. In the absence of UVV, more than three-quarters (77.40%) of the total varicella costs would be attributable to five countries, namely France, Germany, Italy, Spain and the UK. These countries represent the top-five European countries in terms of population size and annual varicella case counts. Annual varicella costs estimated in 2002 for France (148€ million) and Germany (144€ million) before introduction of routine immunization were in line with our findings [5]. Furthermore, France, Germany and UK are among the countries with the highest minimum daily wage (France: €74.92, Germany: €74.9 and UK: €73.36 compared to the median European minimum wage of €42.14). However, it is worth noting that nine countries (Austria, Cyprus, Denmark, Finland, Iceland, Italy, Norway, Sweden and Switzerland) did not have a national minimum wage during the study period [85]. Therefore, the minimum wage was extrapolated using data from the other 22 countries, potentially leading to a bias in the indirect costs for the former countries.

While varicella is commonly perceived as a mild disease, our study shows that it can pose significant economic and caregiver burden. It is important to consider strategies to reduce the clinical and economic burden of varicella across Europe. As of January 2021, nine European countries had UVV [13]. Countries that implemented UVV experienced a significant decline in the varicella burden [3, 11] and several studies indicated that varicella vaccination is cost-saving if productivity losses are considered [10, 11, 83, 86–88]. For example, in Spain, the country with the highest proportion of direct costs in this study (Fig. 3), it was demonstrated that routine vaccination in children aged 1–2 years is cost-saving regardless of indirect costs [25]. European countries may consider UVV depending upon the country-specific burden of disease and the cost-effectiveness of varicella vaccination in their country. Recently published studies have demonstrated the cost-effectiveness of implementing UVV in UK, Italy, Norway and Turkey [9, 88–90]. A budget impact analysis for Denmark [26] showed that the cost of implementation of two-dose UVV program with Varivax® was €5.29–6.76 million depending on vaccination coverage and vaccine cost. This is less than the annual cost of €7.23 million associated with the disease in Denmark [91]. Some disease models have predicted an increase in HZ after implementing UVV programs due to a reduction in circulating VZV [92–94]. However, several epidemiological studies as well as recent modeling studies showed no evidence of an increase in HZ incidence post-UVV [89, 95–100].

The main limitation of our study concerns the lack of availability of several unit cost and utilization input parameters, such as the cost of tests or procedures, which were not included in the final model. Moreover, the extrapolation of data from countries with available information to other countries lacking such data might have introduced a bias due to heterogeneity of health resource costs and resource utilization rates that occurs across countries. Health-economic results are usually not directly comparable from one country to the other and need specific adjustments [9, 86, 101]. Similarly, the disease burden parameters may also have been under- or overestimated since similar imputation methods have been used in the Riera-Montes et al. study [4], and some of the data sources might not be representative of the current situation. However, we have tried to address these multiple sources of uncertainty by adjusting unit costs for inflation and PPP, and conducting stochastic modeling [101]. Adjusting the unit costs of the tradeable health care costs (OTC and prescription medication) using exchange rates resulted in a reduction of total costs of only 0.1% of total costs, thereby suggesting the robustness of our primary approach of adjusting all unit costs by PPP [17, 102, 103]. Another limitation is that potential long-term complications and sequelae such as congenital varicella syndrome or severe cutaneous scarring were not included in this cost-analysis due to lack of data on such costs, leading to possible underestimation of total disease burden. Long-term sequelae are reported in 0.4–3.1% of patients hospitalized due to varicella infections [3]. Additionally, we chose to be conservative with respect to indirect costs since we considered minimum wages, whereas several other economic studies have based their cost estimation on average wages instead [19]. Although small, the unit cost of premature burial [104] estimated at €1008 and the annual premature burial costs without UVV in Europe estimated at €80,672 (representing an increase of 0.01% of total costs), were not accounted for in our study resulting in conservative estimates of total costs of varicella. Lastly, although burden of disease input parameters were stratified by age, our model did not consider potential age-related differences in the unit parameters. Nevertheless, the uncertainty among input parameters was accounted for by combining SLR and stochastic approaches. Furthermore, our study is aligned with previous cost-effectiveness analyses that emphasize the importance of capturing indirect costs to provide a comprehensive picture of the economic burden associated with varicella [11, 105].

It is important to state that we have not investigated in our study the burden due to the loss in quality of life (QoL) nor the quality-adjusted life-year (QALY) although the latter is widely used as measure of the incremental effect in economic evaluations of vaccination. On the basis of Health Utilities Index mark 2 (HUI2) ratings, Brisson et al. [35] found 0.004 and 0.005 QALY loss per episode of varicella, for children younger than 14 years and individuals 15 years and older, respectively. Bilcke et al. [38] found a slightly higher QALY loss, of 0.004 or 0.010, depending whether the patient consulted a physician or not.

## Conclusions

Varicella has a significant public health impact. Its economic burden in absence of UVV is considerable in Europe, mainly owing to high disease incidence and associated health care resource use and caregiver burden including work productivity losses. Assessing the economic burden of a disease is essential for prioritizing healthcare interventions among competing vaccine-preventable diseases, and this analysis underscores the need for more country-specific evaluations to allow informed decision making. These country specific economic data could be used for potential country-specific cost-effectiveness evaluations that would be valuable to support national immunization policy decisions.

## Supplementary Information

**Additional file 1.** Search strategy Systematic literature review.

**Additional file 2.** Data management.

**Additional file 3.** Estimated disease burden in absence of UVV: annual varicella cases, deaths and resource utilization by country and age group.

**Additional file 4.** Cost model.

**Additional file 5.** Varicella unit cost and utilization outcomes (SLR outcomes).

## Data Availability

The datasets supporting the conclusions of this article (including unit cost and utilization outcomes) are included within the article and its additional files.

## Abbreviations

ECDC: European Center for Disease Prevention and Control
ER: Emergency Room
GDP: Gross Domestic Product
HZ: Herpes Zoster
ICU: Intensive Care Unit
OTC: Over-the-counter
PPP: Purchasing Power Parities
PRISMA: Preferred Reporting Items for Systematic Reviews and Meta-Analyses
SLR: Systematic Literature Review
UK: United Kingdom
UVV: Universal Varicella Vaccination
WHO: World Health Organization
