# Supplementary material for: Economic burden of varicella in Europe in the absence of universal varicella vaccination
Source: BMC Public Health. 2021 Dec 21;21:2312. doi: 10.1186/s12889-021-12343-x (PMC8690977; doi:10.1186/s12889-021-12343-x)
Supplement: Supplementary file 1 — Additional file 1. Search strategy Systematic literature review. [file 12889_2021_12343_MOESM1_ESM.docx]

**Additional file 1: Search strategy Systematic literature review**

The full search string used for the Systematic Literature Review was the following:

("herpesvirus 3, human"[MeSH Terms] OR "human herpesvirus 3"[All Fields] OR "varicella"[All Fields] OR "chickenpox"[MeSH Terms] OR "chickenpox"[All Fields]) OR ("herpesvirus 3, human"[MeSH Terms] OR "human herpesvirus 3"[All Fields] OR ("varicella"[All Fields] AND "zoster"[All Fields] AND "virus"[All Fields]) OR "varicella zoster virus"[All Fields]) OR ("varicella zoster virus infection"[MeSH Terms] OR ("varicella"[All Fields] AND "zoster"[All Fields] AND "virus"[All Fields] AND "infection"[All Fields]) OR "varicella zoster virus infection"[All Fields]) OR ("herpesvirus 3, human"[MeSH Terms] OR "human herpesvirus 3"[All Fields] OR "vzv"[All Fields])

**AND**

((((((((((((((("economics"[MeSH Terms] OR "economics"[All Fields] OR "economic"[All Fields]) OR "economics"[Subheading]) OR (("economics"[Subheading] OR "economics"[All Fields] OR "cost"[All Fields] OR "costs and cost analysis"[MeSH Terms] OR ("costs"[All Fields] AND "cost"[All Fields] AND "analysis"[All Fields]) OR "costs and cost analysis"[All Fields]) OR ("costs and cost analysis"[MeSH Terms] OR ("costs"[All Fields] AND "cost"[All Fields] AND "analysis"[All Fields]) OR "costs and cost analysis"[All Fields] OR "costs"[All Fields]))) OR ("Price"[All Fields] OR "prices"[All Fields])) OR ("budgets"[MeSH Terms] OR "budgets"[All Fields] OR "budget"[All Fields])) OR (Fee[All Fields] OR ("economics"[Subheading] OR "economics"[All Fields] OR "fees"[All Fields] OR "fees and charges"[MeSH Terms] OR ("fees"[All Fields] AND "charges"[All Fields]) OR "fees and charges"[All Fields]))) OR ("health expenditures"[MeSH Terms] OR ("health"[All Fields] AND "expenditures"[All Fields]) OR "health expenditures"[All Fields] OR "expenditure"[All Fields])) OR ("presenteeism"[MeSH Terms] OR "presenteeism"[All Fields])) OR ("absenteeism"[MeSH Terms] OR "absenteeism"[All Fields])) OR (("efficiency"[MeSH Terms] OR "efficiency"[All Fields] OR "productivity"[All Fields]) AND loss[All Fields])) OR (Loss[All Fields] AND ("economics"[MeSH Terms] OR "economics"[All Fields] OR "production"[All Fields]))) OR ("outpatients"[MeSH Terms] OR "outpatients"[All Fields] OR "outpatient"[All Fields])) OR "ambulatory care"[MeSH Terms]) OR ("hospitalisation"[All Fields] OR "hospitalization"[MeSH Terms] OR "hospitalization"[All Fields])) OR ("salaries and fringe benefits"[MeSH Terms] OR ("salaries"[All Fields] AND "fringe"[All Fields] AND "benefits"[All Fields]) OR "salaries and fringe benefits"[All Fields] OR "wage"[All Fields])) OR (("work"[MeSH Terms] OR "work"[All Fields] OR "working"[All Fields]) AND hour[All Fields])

**AND**

(("european union"[MeSH Terms] OR ("european"[All Fields] AND "union"[All Fields]) OR "european union"[All Fields]) OR ("europe"[MeSH Terms] OR "europe"[All Fields])) OR (("austria"[MeSH Terms] OR "austria"[All Fields]) OR ("belgium"[MeSH Terms] OR "belgium"[All Fields]) OR ("bulgaria"[MeSH Terms] OR "bulgaria"[All Fields]) OR ("croatia"[MeSH Terms] OR "croatia"[All Fields]) OR ("cyprus"[MeSH Terms] OR "cyprus"[All Fields]) OR ("czech republic"[MeSH Terms] OR ("czech"[All Fields] AND "republic"[All Fields]) OR "czech republic"[All Fields]) OR ("denmark"[MeSH Terms] OR "denmark"[All Fields]) OR ("estonia"[MeSH Terms] OR "estonia"[All Fields]) OR ("finland"[MeSH Terms] OR "finland"[All Fields]) OR ("france"[MeSH Terms] OR "france"[All Fields]) OR ("germany"[MeSH Terms] OR "germany"[All Fields]) OR ("greece"[MeSH Terms] OR "greece"[All Fields]) OR ("hungary"[MeSH Terms] OR "hungary"[All Fields]) OR ("ireland"[MeSH Terms] OR "ireland"[All Fields]) OR ("italy"[MeSH Terms] OR "italy"[All Fields]) OR ("latvia"[MeSH Terms] OR "latvia"[All Fields]) OR ("lithuania"[MeSH Terms] OR "lithuania"[All Fields]) OR ("luxembourg"[MeSH Terms] OR "luxembourg"[All Fields]) OR ("malta"[MeSH Terms] OR "malta"[All Fields]) OR ("netherlands"[MeSH Terms] OR "netherlands"[All Fields]) OR ("poland"[MeSH Terms] OR "poland"[All Fields]) OR ("portugal"[MeSH Terms] OR "portugal"[All Fields]) OR ("romania"[MeSH Terms] OR "romania"[All Fields]) OR ("slovakia"[MeSH Terms] OR "slovakia"[All Fields]) OR ("slovenia"[MeSH Terms] OR "slovenia"[All Fields]) OR ("spain"[MeSH Terms] OR "spain"[All Fields]) OR ("sweden"[MeSH Terms] OR "sweden"[All Fields]) OR Kingdom, United[Full Investigator Name] OR ("norway"[MeSH Terms] OR "norway"[All Fields]) OR ("iceland"[MeSH Terms] OR "iceland"[All Fields]) OR ("switzerland"[MeSH Terms] OR "switzerland"[All Fields]))

**Filter**: Humans
