# Supplementary material for: Economic burden of varicella in Europe in the absence of universal varicella vaccination
Source: BMC Public Health. 2021 Dec 21;21:2312. doi: 10.1186/s12889-021-12343-x (PMC8690977; doi:10.1186/s12889-021-12343-x)
Supplement: Supplementary file 2 — Additional file 2. Data management. [file 12889_2021_12343_MOESM2_ESM.docx]

**Additional file 2: Data management**

-Unit cost values expressed in national currencies were converted to euros (using 2018 Eurostat exchange rates^[[1]](#footnote-1)^: Pound Sterling: 1.13; Spanish Peseta: 0.06; Swiss Franc: 0.866).

-Countries were stratified based on minimum daily wage^[[2]](#footnote-2)^: a. high income countries (equal to the median value among available data points (=€42.14/day) or higher: Belgium, Denmark, France, Germany, Netherlands, Norway, Spain, Sweden, Switzerland, UK), and b. in low and middle income countries (below the median value (=€42.14/day): Czechia, Greece, Hungary, Italy, Poland and Romania), and descriptive statistics (mean, min, max) of unit cost /utilization outcomes were obtained.

- For countries for which no data was available for a given unit outcome, two different types of imputation values were generated. For utilization outcomes, we used the mean value from other countries with available data. For cost outcomes, we generated the input by weighting values by purchasing power parities (PPP) (using 2018 Eurostat PPP^[[3]](#footnote-3)^). in the following manner: 1. the PPP-weighted mean of available values for a given item was calculated (each available cost value was multiplied by 1/PPP and, then, their average was calculated). 2. this PPP-weighted mean was multiplied by the PPP of the country for which data was missing.

1. https://ec.europa.eu/eurostat/web/national-accounts/data/database [↑](#footnote-ref-1)
2. <https://ec.europa.eu/eurostat/statistics-explained/index.php/Minimum_wage_statistics#General_overview>, 2018 [↑](#footnote-ref-2)
3. https://ec.europa.eu/eurostat/web/purchasing-power-parities/data/database [↑](#footnote-ref-3)
