# Supplementary material for: Economic burden of varicella in Europe in the absence of universal varicella vaccination
Source: BMC Public Health. 2021 Dec 21;21:2312. doi: 10.1186/s12889-021-12343-x (PMC8690977; doi:10.1186/s12889-021-12343-x)
Supplement: Supplementary file 3 — Additional file 3. Estimated disease burden in absence of UVV: annual varicella cases, deaths and resource utilization by country and age group. [file 12889_2021_12343_MOESM3_ESM.docx]

**Additional file 3: Estimated disease burden in absence of UVV: annual varicella cases, deaths and resource utilization by country and age group**

| **Country** | **Age**  **group** | **Cases** | **Deaths** | **Hospitalizations (Min)** | **Hospitalizations**  **(Max)** | **Outpatient visits (Min)** | **Outpatient visits (Max)** |
| --- | --- | --- | --- | --- | --- | --- | --- |
| Austria | 0-4y | 38820 | 0 | 121 | 147 | 20620 | 22991 |
|  | 5-9y | 35276 | 0 | 42 | 54 | 15177 | 21264 |
|  | 10-14y | 5348 | 0 | 25 | 38 | 6796 | 14592 |
|  | 15-19y | 3072 | 0 | 13 | 22 | 2027 | 4385 |
|  | 20-39y | 4552 | 0 | 70 | 70 | 3175 | 5859 |
|  | 40-64y | 562 | 0 | 0 | 0 | 343 | 562 |
| Belgium | 0-4y | 91112 | 1 | 492 | 492 | 28041 | 28041 |
|  | 5-9y | 27613 | 0 | 22 | 22 | 6733 | 6733 |
|  | 10-14y | 1823 | 0 | 6 | 13 | 1219 | 1219 |
|  | 15-19y | 1386 | 0 | 6 | 13 | 397 | 397 |
|  | 20-39y | 3463 | 0 | 58 | 58 | 1420 | 1420 |
|  | 40-64y | 1461 | 1 | 0 | 38 | 571 | 571 |
| Bulgaria | 0-4y | 26538 | 0 | 82 | 102 | 14097 | 15719 |
|  | 5-9y | 31633 | 0 | 38 | 49 | 13610 | 19068 |
|  | 10-14y | 4146 | 0 | 20 | 29 | 5268 | 11311 |
|  | 15-19y | 2154 | 0 | 9 | 16 | 1421 | 3074 |
|  | 20-39y | 3412 | 0 | 52 | 52 | 2380 | 4392 |
|  | 40-64y | 450 | 0 | 0 | 0 | 275 | 450 |
| Croatia | 0-4y | 16747 | 0 | 53 | 64 | 8895 | 9919 |
|  | 5-9y | 17554 | 0 | 21 | 27 | 7552 | 10582 |
|  | 10-14y | 2526 | 0 | 12 | 18 | 3209 | 6891 |
|  | 15-19y | 1469 | 0 | 6 | 11 | 970 | 2097 |
|  | 20-39y | 2026 | 0 | 31 | 31 | 1413 | 2608 |
|  | 40-64y | 258 | 0 | 0 | 0 | 158 | 258 |
| Cyprus | 0-4y | 4665 | 0 | 14 | 18 | 2478 | 2763 |
|  | 5-9y | 3771 | 0 | 4 | 6 | 1623 | 2273 |
|  | 10-14y | 122 | 0 | 0 | 1 | 155 | 332 |
|  | 15-19y | 105 | 0 | 0 | 1 | 69 | 150 |
|  | 20-39y | 337 | 0 | 5 | 5 | 235 | 432 |
|  | 40-64y | 127 | 0 | 3 | 3 | 79 | 129 |
| Czech Republic | 0-4y | 43218 | 0 | 135 | 163 | 22952 | 25599 |
|  | 5-9y | 54595 | 0 | 64 | 88 | 23488 | 32909 |
|  | 10-14y | 6649 | 0 | 31 | 47 | 8449 | 18141 |
|  | 15-19y | 3180 | 0 | 14 | 23 | 2098 | 4539 |
|  | 20-39y | 5352 | 0 | 82 | 82 | 3733 | 6889 |
|  | 40-64y | 665 | 0 | 0 | 0 | 406 | 665 |
| Denmark | 0-4y | 48022 | 0 | 150 | 180 | 25508 | 28443 |
|  | 5-9y | 11178 | 0 | 13 | 16 | 4808 | 6738 |
|  | 10-14y | 917 | 0 | 3 | 7 | 1166 | 2501 |
|  | 15-19y | 737 | 0 | 3 | 7 | 488 | 1052 |
|  | 20-39y | 1790 | 0 | 29 | 29 | 1251 | 2299 |
|  | 40-64y | 914 | 0 | 19 | 19 | 571 | 933 |
| Estonia | 0-4y | 6198 | 0 | 20 | 23 | 3292 | 3671 |
|  | 5-9y | 6460 | 0 | 8 | 10 | 2779 | 3894 |
|  | 10-14y | 879 | 0 | 4 | 6 | 1117 | 2397 |
|  | 15-19y | 414 | 0 | 2 | 3 | 273 | 591 |
|  | 20-39y | 679 | 0 | 10 | 10 | 474 | 874 |
|  | 40-64y | 79 | 0 | 0 | 0 | 48 | 79 |
| Finland | 0-4y | 28213 | 0 | 89 | 106 | 14984 | 16711 |
|  | 5-9y | 26914 | 0 | 34 | 43 | 11581 | 16223 |
|  | 10-14y | 121 | 0 | 0 | 0 | 154 | 329 |
|  | 15-19y | 113 | 0 | 0 | 0 | 74 | 160 |
|  | 20-39y | 485 | 0 | 0 | 14 | 332 | 623 |
|  | 40-64y | 529 | 1 | 0 | 0 | 335 | 546 |
| France | 0-4y | 524074 | 3 | 2207 | 2922 | 415514 | 507794 |
|  | 5-9y | 195933 | 1 | 143 | 395 | 140360 | 248359 |
|  | 10-14y | 23223 | 0 | 25 | 75 | 15426 | 53782 |
|  | 15-19y | 16060 | 0 | 232 | 232 | 6664 | 14156 |
|  | 20-39y | 27420 | 2 | 560 | 560 | 10872 | 44926 |
|  | 40-64y | 7823 | 4 | 217 | 217 | 6084 | 12169 |
| Germany | 0-4y | 457151 | 1 | 1712 | 1712 | 242809 | 270775 |
|  | 5-9y | 256703 | 0 | 375 | 375 | 110432 | 154721 |
|  | 10-14y | 9060 | 0 | 74 | 74 | 11527 | 24711 |
|  | 15-19y | 7753 | 0 | 41 | 41 | 5101 | 11058 |
|  | 20-39y | 20965 | 0 | 204 | 407 | 14655 | 27071 |
|  | 40-64y | 9550 | 0 | 0 | 0 | 5895 | 9727 |
| Greece | 0-4y | 33165 | 0 | 104 | 104 | 17617 | 19644 |
|  | 5-9y | 40435 | 0 | 67 | 67 | 17400 | 24374 |
|  | 10-14y | 14987 | 0 | 12 | 12 | 19039 | 40894 |
|  | 15-19y | 7420 | 0 | 38 | 54 | 4896 | 10588 |
|  | 20-39y | 7892 | 1 | 102 | 127 | 5508 | 10149 |
|  | 40-64y | 542 | 0 | 0 | 0 | 339 | 564 |
| Hungary | 0-4y | 40728 | 0 | 126 | 154 | 21634 | 24124 |
|  | 5-9y | 40232 | 0 | 47 | 61 | 17310 | 24253 |
|  | 10-14y | 6184 | 0 | 29 | 44 | 7858 | 16873 |
|  | 15-19y | 3367 | 0 | 15 | 24 | 2222 | 4805 |
|  | 20-39y | 4929 | 0 | 76 | 76 | 3438 | 6345 |
|  | 40-64y | 628 | 0 | 0 | 0 | 384 | 628 |
| Iceland | 0-4y | 2438 | 0 | 8 | 9 | 1295 | 1444 |
|  | 5-9y | 1892 | 0 | 2 | 3 | 814 | 1141 |
|  | 10-14y | 0 | 0 | 0 | 0 | 0 | 0 |
|  | 15-19y | 0 | 0 | 0 | 0 | 0 | 0 |
|  | 20-39y | 0 | 0 | 0 | 0 | 0 | 0 |
|  | 40-64y | 0 | 0 | 0 | 0 | 0 | 0 |
| Ireland | 0-4y | 38237 | 0 | 31 | 31 | 20308 | 22647 |
|  | 5-9y | 22974 | 0 | 11 | 11 | 9884 | 13847 |
|  | 10-14y | 250 | 0 | 3 | 3 | 320 | 682 |
|  | 15-19y | 226 | 0 | 0 | 0 | 150 | 323 |
|  | 20-39y | 844 | 0 | 7 | 7 | 589 | 1086 |
|  | 40-64y | 797 | 1 | 6 | 6 | 506 | 813 |
| Italy | 0-4y | 195019 | 1 | 900 | 900 | 181791 | 184368 |
|  | 5-9y | 225981 | 0 | 529 | 529 | 126603 | 140911 |
|  | 10-14y | 26242 | 0 | 149 | 149 | 30138 | 65404 |
|  | 15-19y | 20229 | 0 | 72 | 72 | 13360 | 28865 |
|  | 20-39y | 49943 | 1 | 363 | 363 | 34953 | 64260 |
|  | 40-64y | 25287 | 2 | 157 | 157 | 15917 | 26005 |
| Latvia | 0-4y | 9953 | 0 | 31 | 38 | 5286 | 5895 |
|  | 5-9y | 8225 | 0 | 10 | 13 | 3539 | 4958 |
|  | 10-14y | 1239 | 0 | 6 | 9 | 1574 | 3380 |
|  | 15-19y | 597 | 0 | 3 | 4 | 394 | 853 |
|  | 20-39y | 955 | 0 | 15 | 15 | 666 | 1229 |
|  | 40-64y | 119 | 0 | 0 | 0 | 73 | 119 |
| Lithuania | 0-4y | 14088 | 0 | 43 | 54 | 7483 | 8345 |
|  | 5-9y | 11653 | 0 | 14 | 19 | 5014 | 7025 |
|  | 10-14y | 1627 | 0 | 8 | 12 | 2067 | 4439 |
|  | 15-19y | 989 | 0 | 4 | 7 | 652 | 1411 |
|  | 20-39y | 1367 | 0 | 21 | 21 | 954 | 1760 |
|  | 40-64y | 178 | 0 | 0 | 0 | 109 | 178 |
| Luxembourg | 0-4y | 5071 | 0 | 16 | 19 | 2693 | 3003 |
|  | 5-9y | 1079 | 0 | 1 | 2 | 464 | 650 |
|  | 10-14y | 49 | 0 | 0 | 0 | 62 | 133 |
|  | 15-19y | 42 | 0 | 0 | 0 | 28 | 61 |
|  | 20-39y | 151 | 0 | 2 | 2 | 106 | 195 |
|  | 40-64y | 86 | 0 | 0 | 2 | 54 | 88 |
| Malta | 0-4y | 4129 | 0 | 13 | 16 | 2193 | 2445 |
|  | 5-9y | 458 | 0 | 0 | 1 | 197 | 276 |
|  | 10-14y | 56 | 0 | 0 | 0 | 72 | 154 |
|  | 15-19y | 48 | 0 | 0 | 0 | 32 | 69 |
|  | 20-39y | 180 | 0 | 3 | 3 | 126 | 231 |
|  | 40-64y | 72 | 0 | 2 | 2 | 45 | 74 |
| Netherlands | 0-4y | 139955 | 0 | 127 | 161 | 26321 | 50497 |
|  | 5-9y | 31090 | 0 | 16 | 18 | 5485 | 14246 |
|  | 10-14y | 193 | 0 | 2 | 3 | 503 | 1740 |
|  | 15-19y | 210 | 0 | 1 | 2 | 335 | 964 |
|  | 20-39y | 766 | 0 | 26 | 26 | 978 | 2127 |
|  | 40-64y | 893 | 1 | 12 | 18 | 705 | 1352 |
| Norway | 0-4y | 39781 | 0 | 123 | 150 | 21129 | 23562 |
|  | 5-9y | 17388 | 0 | 23 | 26 | 7480 | 10480 |
|  | 10-14y | 859 | 0 | 3 | 6 | 1092 | 2342 |
|  | 15-19y | 686 | 0 | 3 | 6 | 454 | 979 |
|  | 20-39y | 1749 | 0 | 28 | 28 | 1223 | 2247 |
|  | 40-64y | 824 | 1 | 17 | 17 | 515 | 841 |
| Poland | 0-4y | 170271 | 1 | 531 | 645 | 74548 | 74548 |
|  | 5-9y | 158250 | 1 | 184 | 246 | 84015 | 84015 |
|  | 10-14y | 30450 | 0 | 147 | 203 | 17013 | 17013 |
|  | 15-19y | 15212 | 0 | 74 | 111 | 3646 | 3646 |
|  | 20-39y | 21031 | 0 | 329 | 329 | 8456 | 8456 |
|  | 40-64y | 1235 | 0 | 0 | 0 | 1157 | 1157 |
| Portugal | 0-4y | 54017 | 0 | 166 | 204 | 28691 | 31994 |
|  | 5-9y | 27934 | 0 | 34 | 43 | 12017 | 16838 |
|  | 10-14y | 1403 | 0 | 5 | 10 | 1784 | 3827 |
|  | 15-19y | 1184 | 0 | 6 | 11 | 784 | 1690 |
|  | 20-39y | 2932 | 0 | 48 | 48 | 2050 | 3766 |
|  | 40-64y | 1783 | 0 | 37 | 37 | 1114 | 1820 |
| Romania | 0-4y | 70466 | 1 | 218 | 268 | 10905 | 10905 |
|  | 5-9y | 98312 | 0 | 121 | 151 | 14613 | 14613 |
|  | 10-14y | 13345 | 0 | 63 | 95 | 10533 | 10533 |
|  | 15-19y | 7245 | 0 | 32 | 53 | 5265 | 5265 |
|  | 20-39y | 9732 | 0 | 150 | 150 | 4429 | 4429 |
|  | 40-64y | 1239 | 0 | 0 | 0 | 556 | 556 |
| Slovakia | 0-4y | 26958 | 0 | 84 | 101 | 14320 | 15967 |
|  | 5-9y | 24246 | 0 | 29 | 38 | 10433 | 14614 |
|  | 10-14y | 2367 | 0 | 11 | 16 | 3008 | 6459 |
|  | 15-19y | 1496 | 0 | 8 | 11 | 988 | 2136 |
|  | 20-39y | 3131 | 0 | 47 | 47 | 2182 | 4033 |
|  | 40-64y | 501 | 0 | 0 | 0 | 323 | 512 |
| Slovenia | 0-4y | 12049 | 0 | 37 | 46 | 6399 | 7136 |
|  | 5-9y | 7758 | 0 | 9 | 12 | 3338 | 4677 |
|  | 10-14y | 262 | 0 | 1 | 2 | 333 | 715 |
|  | 15-19y | 205 | 0 | 1 | 2 | 135 | 292 |
|  | 20-39y | 676 | 0 | 10 | 10 | 471 | 871 |
|  | 40-64y | 381 | 0 | 7 | 7 | 240 | 390 |
| Spain | 0-4y | 229031 | 1 | 493 | 1064 | 174901 | 174901 |
|  | 5-9y | 177241 | 0 | 131 | 288 | 79531 | 79531 |
|  | 10-14y | 7688 | 0 | 22 | 76 | 18289 | 18289 |
|  | 15-19y | 5886 | 0 | 20 | 20 | 3894 | 8399 |
|  | 20-39y | 18488 | 3 | 235 | 347 | 12886 | 23754 |
|  | 40-64y | 12283 | 2 | 173 | 276 | 7763 | 12594 |
| Sweden | 0-4y | 81746 | 1 | 52 | 181 | 43419 | 48416 |
|  | 5-9y | 31588 | 0 | 39 | 39 | 13590 | 19039 |
|  | 10-14y | 1579 | 0 | 7 | 7 | 2008 | 4307 |
|  | 15-19y | 1161 | 0 | 5 | 11 | 768 | 1656 |
|  | 20-39y | 3249 | 0 | 53 | 53 | 2272 | 4174 |
|  | 40-64y | 1504 | 1 | 31 | 31 | 940 | 1535 |
| Switzerland | 0-4y | 31958 | 0 | 100 | 121 | 16972 | 18929 |
|  | 5-9y | 50317 | 0 | 60 | 77 | 21648 | 30332 |
|  | 10-14y | 303 | 0 | 0 | 4 | 384 | 826 |
|  | 15-19y | 293 | 0 | 0 | 0 | 194 | 418 |
|  | 20-39y | 1221 | 0 | 23 | 23 | 859 | 1560 |
|  | 40-64y | 1058 | 1 | 0 | 0 | 654 | 1100 |
| UK | 0-4y | 512627 | 4 | 1528 | 2128 | 151553 | 185394 |
|  | 5-9y | 192159 | 1 | 318 | 673 | 64466 | 124309 |
|  | 10-14y | 14727 | 0 | 57 | 118 | 12867 | 24786 |
|  | 15-19y | 11919 | 0 | 99 | 202 | 9013 | 13428 |
|  | 20-39y | 37045 | 4 | 473 | 998 | 37308 | 44665 |
|  | 40-64y | 20102 | 11 | 127 | 211 | 4645 | 14147 |
