# Supplementary material for: Economic burden of varicella in Europe in the absence of universal varicella vaccination
Source: BMC Public Health. 2021 Dec 21;21:2312. doi: 10.1186/s12889-021-12343-x (PMC8690977; doi:10.1186/s12889-021-12343-x)
Supplement: Supplementary file 4 — Additional file 4. Cost model. [file 12889_2021_12343_MOESM4_ESM.docx]

**Additional file 4: Cost model**

The overall annual direct costs of varicella (Cost_direct) was estimated as follows:

Cost_direct=∑i ∑j n_Hospij*c_Hospi*u_Hospi + n_Outij*c_Outi + n_Outij*c_Presci*u_Presci + n_Casesij*c_OTCi*u_OTCi

where i represents every country and j every age group; n_Hosp is the number of hospitalizations, c_Hosp the unit cost of a 1-day hospitalization, and u_Hosp the length of hospitalization; n_Out is the number of doctor (outpatient) visits and c_Out the unit cost of an outpatient visit; ; c_Presc is the unit cost of prescription medications and u_Presc is the utilization of OTC medications ; n_Cases is the number of Varicella cases, c_OTC is the unit cost of OTC medications and u_OTC is the utilization of OTC medications.

The overall annual indirect costs of varicella (Cost_indirect) were determined using the human capital approach, separately for children (≤19 years) and adults (>19 years) patients:

Cost_indirect_children=∑i ∑j c_Wagei*Empi * (n_Outij*u_Care i + n_Hospij*u_Hospi)

C_indirect_adults=∑i ∑j c_Wagei *Empi* (n_Outij*u_Patii + n_Hospij*u_Hospii + n_Deathij*WorkYri)

where c_Wage represents the minimal daily wage^[[1]](#footnote-1)^, Emp the employment rate among the 20-64 years population^[[2]](#footnote-2)^, u_Care the number of work days lost by caregiver (assuming one caregiver per child outpatient per day), u_Pati the number of work days lost by the adult outpatient, n_Death is the number of varicella-related deaths and WorkYr the number of work days lost because of premature death ranging from 1 day to the number of working days in 2018^[[3]](#footnote-3)^

To account for the uncertainty of the input parameters, and assuming that all values falling within the min-max range were equally probable, a uniform distribution (min-max) was assigned to each parameter in our model. The parameter distributions were then combined through stochastic modeling; a total of 10,000 samples were drawn (10,000 iterations), and the mean and min-max output of the output values were used to estimate population-level direct costs (outpatient visits, hospitalizations, prescription/OTC medications) and indirect costs (work loss caregivers/patients/deaths) for the 31 European countries. The distribution of costs by country, age group and healthcare resource (outpatient visits/hospitalizations/prescription medication/OTC medication/work loss caregivers/work loss patients) was also described. The mean (direct/indirect/total) costs per varicella case were calculated by dividing the population-level costs by the total number of annual varicella cases in Europe.

1. https://ec.europa.eu/eurostat/statistics-explained/index.php/Minimum_wage_statistics#General_overview [↑](#footnote-ref-1)
2. <https://ec.europa.eu/eurostat/web/products-datasets/-/tesem010> [↑](#footnote-ref-2)
3. https://ec.europa.eu/eurostat/cros/content/euro-area-and-eu-working-days-build-calendar-adjustment-regressor_en [↑](#footnote-ref-3)
