# Supplementary material for: Economic burden of varicella in Europe in the absence of universal varicella vaccination
Source: BMC Public Health. 2021 Dec 21;21:2312. doi: 10.1186/s12889-021-12343-x (PMC8690977; doi:10.1186/s12889-021-12343-x)
Supplement: Supplementary file 5 — Additional file 5. Varicella unit cost and utilization outcomes (SLR outcomes). [file 12889_2021_12343_MOESM5_ESM.docx]

**Additional file 5: Varicella unit cost and utilization outcomes (SLR outcomes)**

| \| **Country** \| **Cost of physician visit (EUR)** \| **Cost of 1 day hospitalization (EUR)** \| **Length hospitalization (days)** \| **Cost of OTC medications (EUR)** \| **Utilization OTC medication(%)** \| **Cost of prescribed medication (EUR)** \| **Utilization of prescribed medication (%)** \| **Work lost by caregiver (days)** \| **Work lost by patient (days)** \| \| --- \| --- \| --- \| --- \| --- \| --- \| --- \| --- \| --- \| --- \| \| Belgium \|  \| 649.68 \| 3-4.7 \|  \|  \|  \|  \| 2 \|  \| \| Czechia \|  \|  \| 7 \|  \|  \|  \|  \|  \|  \| \| Denmark \|  \|  \| 2 \|  \|  \|  \|  \|  \|  \| \| France \| 21.9-31.21 \| 437.38-720.82 \| 1.7-5 \|  \|  \|  \|  \| 0.6-4.7 \| 4.7-10.9 \| \| Germany \| 12.53-27.35 \| 268.64-598.59 \| 3-5.3 \| 14.34 \|  \|  \|  \| 0.7-6.6 \| 5.3-18.72 \| \| Greece \|  \|  \| 5.62 \|  \|  \|  \|  \|  \|  \| \| Hungary \| 9.55 \| 97.03 \| 3.6 \| 0.4 \| 96 \| 31.57 \| 9.3 \| 2.5 \|  \| \| Italy \| 20.99-37.05 \| 413.01-629 \| 4-7.9 \| 4.84 \| 100 \| 18.8-23.97 \| 64-100 \| 0.6-4.98 \| 2.6-11 \| \| Netherlands \| 22.73 \| 404 \| 6.7 \|  \|  \|  \| 47.7-53.6 \| 0.3-1.65 \| 14.35 \| \| Norway \| 38.02 \| 1304.44 \| 3 \|  \|  \|  \|  \|  \|  \| \| Poland \| 52.29 \| 155.35 \| 4.73-7 \| 0.55 \| 80 \| 9.45 \| 80 \| 2.71 \|  \| \| Romania \|  \|  \| 6.16 \|  \|  \|  \|  \|  \|  \| \| Spain \| 4.52-72.41 \| 128.85-895.86 \| 4-9 \| 2.05-12.15 \| 100 \| 18.81-25.77 \| 87-100 \| 0.97 \| 10.5 \| \| Sweden \|  \|  \| 7.2 \|  \|  \|  \|  \| 3.5 \|  \| \| Switzerland \| 22.81 \| 510.13 \| 8 \| 15.86 \|  \|  \|  \|  \| 1.44 \| \| UK \| 27.47-62.3 \| 307.04-752.71 \| 2.7-6.4 \|  \|  \| 2.8-11.51 \|  \| 0.6 \| 5.7 \| |
| --- | --- | --- | --- | --- | --- | --- | --- | --- | --- | --- | --- | --- | --- | --- | --- | --- | --- | --- | --- | --- | --- | --- | --- | --- | --- | --- | --- | --- | --- | --- | --- | --- | --- | --- | --- | --- | --- | --- | --- | --- | --- | --- | --- | --- | --- | --- | --- | --- | --- | --- | --- | --- | --- | --- | --- | --- | --- | --- | --- | --- | --- | --- | --- | --- | --- | --- | --- | --- | --- | --- | --- | --- | --- | --- | --- | --- | --- | --- | --- | --- | --- | --- | --- | --- | --- | --- | --- | --- | --- | --- | --- | --- | --- | --- | --- | --- | --- | --- | --- | --- | --- | --- | --- | --- | --- | --- | --- | --- | --- | --- | --- | --- | --- | --- | --- | --- | --- | --- | --- | --- | --- | --- | --- | --- | --- | --- | --- | --- | --- | --- | --- | --- | --- | --- | --- | --- | --- | --- | --- | --- | --- | --- | --- | --- | --- | --- | --- | --- | --- | --- | --- | --- | --- | --- | --- | --- | --- | --- | --- | --- | --- | --- | --- | --- | --- | --- | --- | --- | --- | --- |
